# Two Wheat Glutathione Peroxidase Genes Whose Products Are Located in Chloroplasts Improve Salt and H_2_O_2_ Tolerances in *Arabidopsis*

**DOI:** 10.1371/journal.pone.0073989

**Published:** 2013-10-02

**Authors:** Chao-Zeng Zhai, Lei Zhao, Li-Juan Yin, Ming Chen, Qing-Yu Wang, Lian-Cheng Li, Zhao-Shi Xu, You-Zhi Ma

**Affiliations:** 1 Institute of Crop Science, Chinese Academy of Agricultural Sciences/National Key Facility for Crop Gene Resources and Genetic Improvement, Key Laboratory of Biology and Genetic Improvement of Triticeae Crops, Ministry of Agriculture, Beijing, China; 2 College of Plant Science, Jilin University, Changchun, China; University of Nottingham, United Kingdom

## Abstract

Oxidative stress caused by accumulation of reactive oxygen species (ROS) is capable of damaging effects on numerous cellular components. Glutathione peroxidases (GPXs, EC 1.11.1.9) are key enzymes of the antioxidant network in plants. In this study, *W69* and *W106*, two putative GPX genes, were obtained by *de novo* transcriptome sequencing of salt-treated wheat (*Triticum aestivum*) seedlings. The purified His-tag fusion proteins of W69 and W106 reduced H_2_O_2_ and *t*-butyl hydroperoxide (t-BHP) using glutathione (GSH) or thioredoxin (Trx) as an electron donor *in vitro,* showing their peroxidase activity toward H_2_O_2_ and toxic organic hydroperoxide. GFP fluorescence assays revealed that W69 and W106 are localized in chloroplasts. Quantitative real-time PCR (Q-RT-PCR) analysis showed that two GPXs were differentially responsive to salt, drought, H_2_O_2,_ or ABA. Isolation of the *W69* and *W106* promoters revealed some *cis*-acting elements responding to abiotic stresses. Overexpression of *W69* and *W106* conferred strong tolerance to salt, H_2_O_2_, and ABA treatment in *Arabidopsis*. Moreover, the expression levels of key regulator genes (*SOS1, RbohD* and *ABI1/ABI2*) involved in salt, H_2_O_2_ and ABA signaling were altered in the transgenic plants. These findings suggest that *W69* and *W106* not only act as scavengers of H_2_O_2_ in controlling abiotic stress responses, but also play important roles in salt and ABA signaling.

## Introduction

In higher plant cells, several metabolic processes (e.g. photosynthesis and respiration) and adverse environmental conditions (e.g. high-salt, drought, mechanical stimulation, chemical toxicity, pathogen infections and extreme temperatures) promote generation of reactive oxygen species (ROS), including hydroxyl radicals (OH), superoxide radicals (O_2_^·−^), and hydrogen peroxide (H_2_O_2_) [1]–[5]. ROS are capable of causing damage to membrane lipids, proteins and nucleic acids and these highly reactive molecules are believed to be the major contributing factors in causing rapid cell damage [6]. Exogenous H_2_O_2_ induces the expression of defense genes as well as initiating programmed cell death in plants [7], [8]. The steady-state level of ROS is mainly determined by the activity of antioxidant system in plant cells [9]. Several endogenous antioxidant enzymes, such as superoxide dismutase (SOD), glutathione peroxidase (GPX), catalase (CAT), ascorbate peroxidase (APX), and glutathione reductase (GR) play important roles in protecting plants cell from oxidative injury [10]. GPX is a key enzyme for scavenging H_2_O_2_ in plant cells [11].

As an antioxidant enzyme, GPX in mammals reduces H_2_O_2_ and organic hydroperoxides to water and correspondingly alcohols using glutathione (GSH) to protect cells from oxidative damage [12], [13]. Based on their amino acid sequences, substrate specificity, and tissue localization, the GPX family in mammals can be divided into five classes, cytosolic GPX (GPX1), gastro-intestinal GPX (GPX2), plasma GPX (GPX3), phospholipid hydroperoxide GPX (PHGPX, also called GPX4), and seleno-independent epididymis GPX (GPX5) [12], [14]. Among GPX isoforms, vertebrate GPX5 proteins lack the selenocysteine residue replaced by a cysteine residue in the catalytic site [15], [16]. The selenocysteine residue in its presumed catalytic site is important for the catalytic activity of GPXs, but the replacement of selenocysteine by cysteine greatly reduces the activity of enzymes in animals [17]. PHGPX is an antioxidant selenoenzyme present in a variety of adult and embryonic tissues. It is the principal basis of a defense system that intimately participates in the repair of disrupted biomembranes by interacting directly with peroxidized phospholipids in biomembranes [18]–[21].

The plant GPX family shares highest sequence homology to animal PHGPXs, rather than to any of the other members of the animal GPX family [22]–[26]. These proteins from plants possess a Cys residue, rather than selenoCys, in their presumed catalytic site, suggesting that GPX in plant is not a Se-dependent protein [27]. However, Amino acid replacement of the catalytic selenoCys by Cys results in a relatively low activity in plants compared to homologous animal GPXs [28]. GPXs are ubiquitously occurring enzymes in subcellular organelles, including the cytosol, nucleus, chloroplast, mitochondria, and peroxisome, where GPXs use GSH or thioredoxin (Trx) as a reducing agent to reduce H_2_O_2_, organic hydroperoxide, and lipid hydroperoxides [2], [12], [29]–[32]. Plant GPX isoenzymes display an obvious preference to Trx [24], [33].

Besides GPXs another group of enzymes capable of scavenging peroxides in plants have been studied extensively. It is known that certain plant glutathione-S-transferase (GST) displayed also strong GPX activity (GST/GPX) toward organic hydroperoxides catalysing their reduction to the less toxic alcohols [34].

Plant GPXs are involved in responses to many abiotic stresses and hormones [27], [35]–[37]. A rice *GPX* gene was strongly expressed in normal tissues of high photosynthetic efficiency as well as in tissues stimulated by oxidative stresses [38]. Overexpression of the tobacco *GST/GPX* gene confers salt and chilling tolerance due to enhanced ROS scavenging and reduces membrane damage in transgenic tobacco plants [10]. It was demonstrated that *Arabidopsis* GPX3 plays dual roles in H_2_O_2_ scavenging and ABA signal transduction [39]. Furthermore, chloroplastic GPXs in *Arabidopsis* play a role in cross talk between photooxidative stress and immune responses [5], [40]. As has been reported, expressing a tomato PHGPx in tobacco could prevent Bax induced cell death, indicating the role of GPX in PCD in plant [41], [42].

Wheat (*Triticum aestivum* L.) is one of the most important cereal crops in the world; however, ROS accumulation in cells induced by environmental stresses can cause severe oxidative damage to wheat growth and grain yield [43]. At present, there is no report on the role of GPXs in protecting wheat plants from oxidative damage. In this study, we isolated two wheat cDNAs encoding GPX proteins for the first time by *de novo* transcriptome assembly technology. The isoenzymatic characteristics, subcellular localization, and transcript accumulation of the recombinant protein when transgenic plants were subjected to different stresses were investigated.

## Results

### Isolation and characterization of *W69* and *W106*

**Figure 1:**
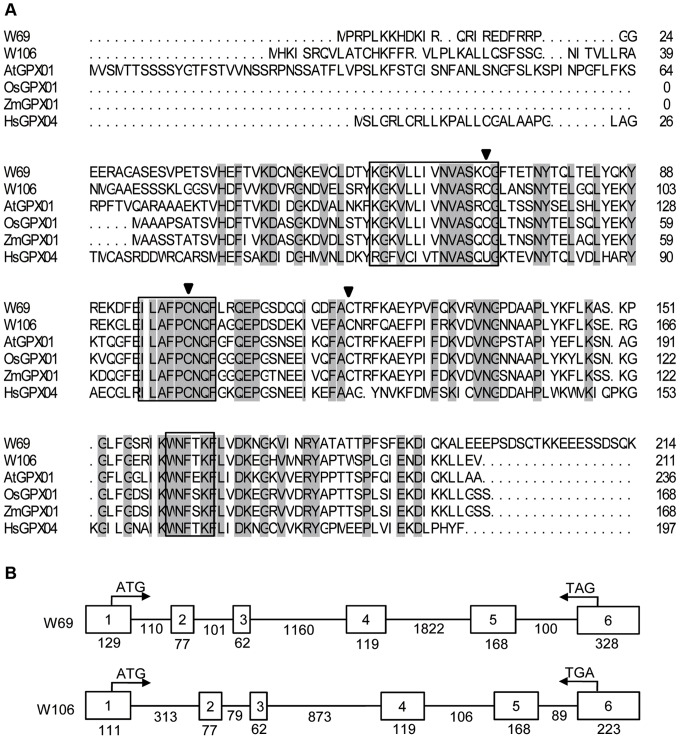
Alignment of W69 and W106 with other GPXs from plants and their exon-intron structures. (A) Alignment of GPX protein sequences with other species: *Oryza sativa* (OsGPX01, accession number: NC008397), *Zea mays* (ZmGPX01, AY542310) and *Homo sapiens* (HsGPX04, P36969). Gray background represents strictly conserved amino acids. Boxed sequences represent highly conserved domains (G1,G2,and G3). The SeCys residues of the mammalian PHGPXs (HsGPX04) are denoted by “U”, the three conserved Cys of these isoenzymes are marked by inverted triangles. (B) Exon-intron structure of the *GPX* homologs. The chromosomal structures of the *GPXs* were constructed by comparing mRNA sequences with their respective genomic sequences. The length of each exon (square) and intron (line) is given. The *W69* gene starts with ATG at position 75 bp in exon 1 and ends with TAG at position 81 bp in exon 6; the *W106* gene starts with ATG at position 56 bp in exon 1 and ends by TGA at position 29 bp in exon 6.

**Figure 2:**
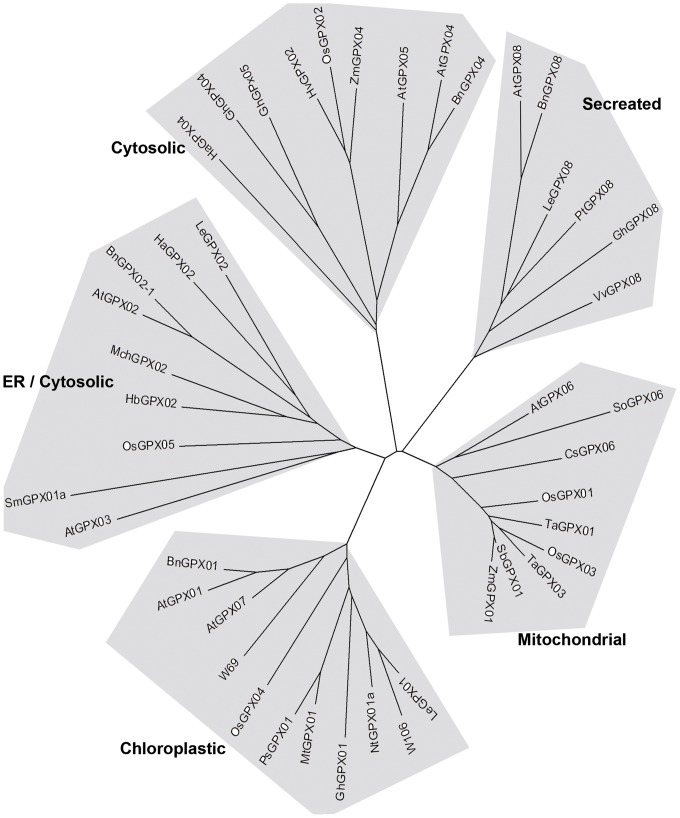
Phylogenetic tree of W69 and W106 with other GPXs from plants. All GPXs proteins were clustered using ClustalX, and the phylogenetic tree was generated by MEGA5 using the neighbor-joining algorithm. In addition to the sequences of W69 and W106, the tree was constructed using sequences from *Arabidopsis thaliana*: AtGPX01, At2g25080; AtGPX02, At2g31570; AtGPX03, At2g43350; AtGPX04, At2g48150; AtGPX05, At3g63080; AtGPX06, At4g11600; AtGPX07, At4g31870; AtGPX08, At1g63460. *Brassica napus*: BnGPx01, ADI58543; BnGPx02-1, AC189433; BnGPx04, ES268656; BnGPx08, TC10360. *Citrus sinensis*: CsGPx06, X66377. *Gossypium hirsutum*: GhGPx01, AI729829; GhGPx04, DV849230; GhGPx05, DW496848; GhGPx08, DW518187. *Helianthus annuus*: HaGPx02, CX945161; HaGPx04, CX947851. *Hevea brasiliensis*: HbGPx02, EC609359. *Hordeum vulgare*, HvGPx02, AK357226. *Lycopersicon esculentum*: LeGPX01, BI934604; LeGPx02, AY301280; LeGPX08, DB689713. *Medicago truncatula*: MtGPx01, AC143339; MchGPx02, AF346906. *Nicotiana tabacum*: NtGPx01a, DV999781. *Oryza sativa ssp japonica cv Nipponbare*: OsGPx01, NC008397; OsGPx02, NC008396; OsGPx03, NC008395; OsGPx04, NC008399; OsGPx05, NC008404. *Pisum sativum*: PsGPx01, AJ000508. *Populus trichocarpa*: PtGPX08, EEE84340. *Selaginella moellendorffii*: SmGPx01a, DN838361. *Sorghum bicolor*: SbGPx01, AAT42166; *Spinacia oleracea*: SoGPx06, D63425. *Triticum aestivum*: TaGPX01, AJ010455; TaGPx03, JP214946. *Vitis vinifera*: VvGPX08, EE064395. *Zea mays*: ZmGPx01, AY542310; ZmGPX04, EU971245.

We generated an extensive expressed gene catalog for salt-treated wheat using Illumina mRNA Sequencing technology and *de novo* assembly (unpublished data). Two up-regulated putative GPX cDNA fragments, designated as *W69* (GenBank Accession No. KF031945) and *W106* (GenBank Accession No. KF031946), were selected from the total transcript-derived assembled unigenes. Full-length cDNA of *W69* and *W106* were cloned successfully from wheat cDNA by reverse transcription PCR (RT-PCR) using special primers sets. The predicted W69 protein consists of 214 amino acids with a calculated molecular mass of 21.3 kD, and W106 protein consists of 211 amino acids and calculated molecular mass of 19.2 kD. Multiple sequence alignment of W69 and W106 with other reported GPXs from animals and plants showed that some motifs of GPXs were conserved among different species (Fig. 1A). Three completely conserved motifs GKVLLIVNVASRCG (GPX signature 1), LAFPCNQ (GPX signature 2), and WNF(S/T)KF) existed within most plant and mammalian GPX sequences. Cysteine residues coded by UGU located in the “GPX signature 1” are the specific structure of selenium-independent plant GPXs, whereas selenium-dependent HsGPXs from mammals possess a seleno-cysteine residue coded by UGA (Fig. 1A). A phylogenetic tree of GPXs from plants revealed that GST family can be classified into five classes. W69 and W106 belong to one group located to chloroplasts (Fig. 2B).

Isolation of the genomic sequences of the wheat *GPX*s revealed both *W69* and *W106* comprised six exons and five introns (Fig. 1B). All of the exon/intron splice junctions in W69 DNA sequence conform to the canonical GT/AG boundary [44], intron 5 of *W106* gene has a GT/GC sequence.

To gain insight into the mechanism responsible for transcriptional regulation, we isolated a 1,862 bp promoter region upstream of the *W69* ATG start codon, and 1,600 bp of W106 from genomic DNA using a PCR strategy. We searched for putative *cis*-acting elements in the promoter regions using the database Plant *cis*-acting Elements, PLACE (http://www.dna.affrc.go.jp/PLACE/) (Table 1). A number of regulatory elements responsive to drought, salt, low-temperature, and ABA were recognized, including ABRE, DRE, DRE/CRT, and DPBF binding sequences. In addition, a gibberellin responsive element (GARE) and an ethylene responsive element (ERE) were identified. More importantly, the *W69* and *W106* promoter regions have antioxidant-responsive elements, ARE, which was found in plant and human promoters (Table 1).

**Table 1:** Putative *cis*-acting elements in the wheat *W69 and W106* promoters.

Element	Promoter	sequence	Function	Reference
**ARE**	W69, W106	AGTGACNNNGC	antioxidant-responsive elements	[76]
**ABRE**	W69, W106	ACGTG(G/T)C	ABA and drought responsive elements	[77]
**CBF**	W69	RYCGAC	Dehydration responsive element	[78]
**DRE**	W69	ACCGAC	ABA and drought responsive elements	[79]
**CRT/DRE**	W69	CCGAC	Drought, high-salt and cold responsive elements	[79]
**DPBF binding site**	W69,W106	ACACNNG	ABA responsive and embryo specification elements	[80]
**GT1GMSCAM4**	W69, W106	GAAAAA	Pathogen and salt responsive elements	[81]
**MYB recognition site**	W69,W106	C/TAACNA/G	ABA and drought responsive elements	[82]
**MYC recognition site**	W69,W106	CATGTG	ABA and drought responsive elements	[83]
**HSE**	W69, W106	AGAAAATTCG	Heat shock responsive element	[84]
**TC-rich repeats**	W69, W106	ATTTTCTTCA	Element involved in defense and stress	[85]
**W-box**	W69, W106	TTGAC	Wound and pathogen responsive elements	[86]
**ERE element**	w106	ATTTCAAA	Ethylene responsive elements	[85]
**GARE-motif**	w106	AAACAGA	Gibberellins responsive element	[87]
**TGACG-motif**	W69, W106	TGACG	MeJA responsive elements	[88]
**AuxRE**	W69	GGTCCAT	Auxin responsive elements	[89]

### Expression patterns of *W69* and *W106* under stress

**Figure 3:**
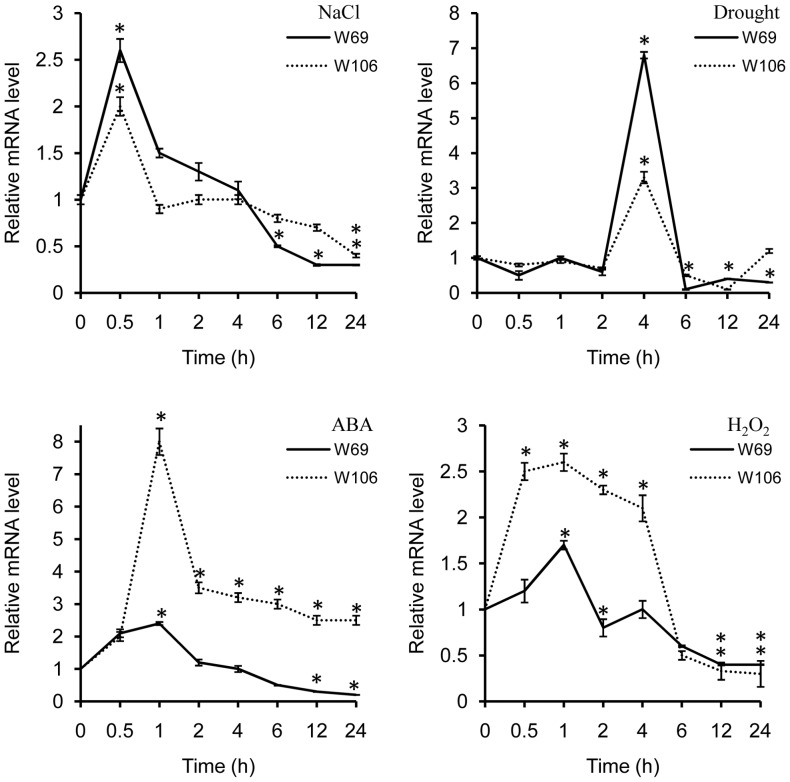
Expression patterns of *W69* and *W106* under various stress conditions, including salinity (A), drought (B), H_2_O_2_ (C), and ABA (D). Total RNA was isolated from leaves of wheat seedlings. The *actin* gene was used as an internal reference. Relative expression of *W69* and *W106* were normalized to the transcript abundances in untreated controls (normalized as 1). Error bars represent standard deviation (SD) among three biological replicates. Asterisks indicate a significant difference (*P<0.05; Student's t-test) relative to untreated control (at 0 hours).

The *W69* and *W106* promoter regions have multiple abiotic stress-responsive elements (Table 1). To assess responses to abiotic stresses, we quantitatively surveyed the transcript expressions of *W69* and *W106* using quantitative real-time PCR (Q-RT-PCR). As shown in Fig. 3, both transcripts began to respond to environmental stress at early stages, but exhibited different expression patterns.

Both *W69* and *W106* were rapidly activated to peak within 0.5 h after salt treatment, then declined to normal levels after 1 h, and were barely detectable within 24 h. Both were also rapidly induced by drought and ABA, reached a maximum level at 4 h and 1 h, respectively, and then declined. *W69* was strongly induced by drought, but only weakly by ABA. In contrast, *W106* was highly activated by ABA, but weakly by drought. In addition, *W106* was immediately activated by H_2_O_2_ and absent after 6 h; however, the transcript of *W69* was barely detectable over a 24 h treatment with H_2_O_2_. These results implied that the GPX subfamily may function mainly in oxidative responses induced by salt, H_2_O_2_ and ABA stresses, but with some divergence in roles between *W69* and *W106*.

### GPX activities of *W69* and *W106 in vitro*

**Figure 4:**
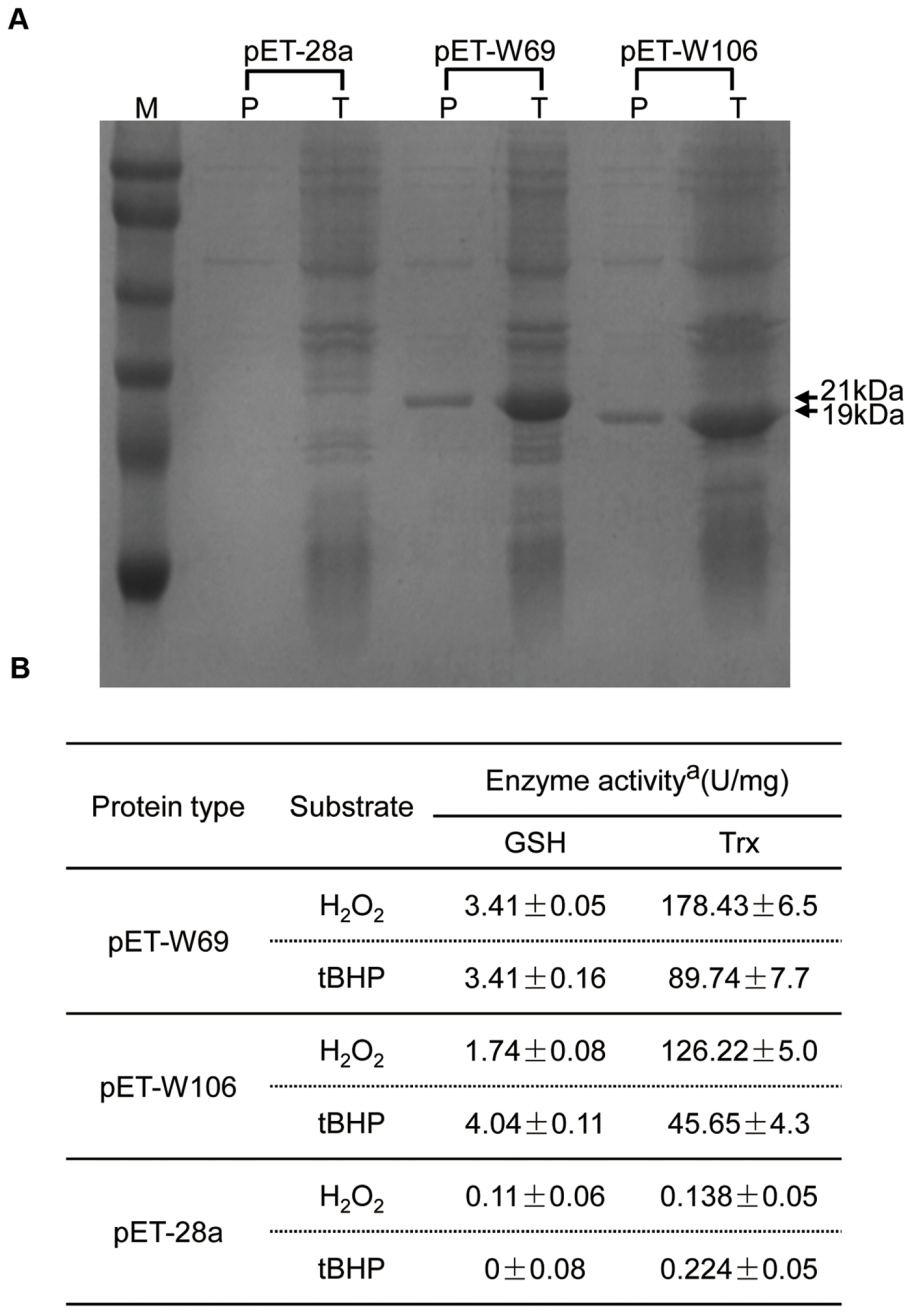
GPX activity analysis in pET-28a, pET-W69 and pET-W106. (A) SDS-PAGE analysis of the expression of recombinant proteins in *E. coli.* The complete protein of the bacteria and purified recombinant protein from soluble crude extract were separated by 12% SDS-PAGE and stained with Coomassie Brilliant Blue. M, protein size marker (16–94 kDa). T, total proteins of the bacteria (10 μg); P, purified recombinant proteins (1 μg) from soluble crude extract. (B) Enzyme activities of the GPX isoenzymes were calculated with H_2_O_2_ and t-BHP as the substrates. Data are means ±SD of three independent experiments.

Overexpression of recombinant GPXs in *E. coli* BL21 cells was observed after IPTG induction. Samples of total proteins and purified soluble protein fractions were separated on SDS-PAGE. W69 (21 kDa) and W106 (19 kDa) were successfully expressed (Fig. 4A).

The purified protein was prepared for enzymatic analysis. Results showed that the recombinant isoenzymes could catalyze H_2_O_2_ and t-BHP using *E. coli* Trx or GSH as electron donors. However, the isoenzymes reduced hydroperoxides with Trx at a higher rate than with GSH, indicating that Trx was likely the favored electron donor (Fig. 4B). In contrast to t-BHP, the isoenzymes exhibited a higher affinity for H_2_O_2_ and GPX activities of W69 were detected at a higher level than W106 utilizing Trx, which is the main electron donor during redox reactions. As expected, the pET-28A-transformed control had no obvious GPX activity (Fig. 4B).

### Chloroplast localization of W69 and W106

**Figure 5:**
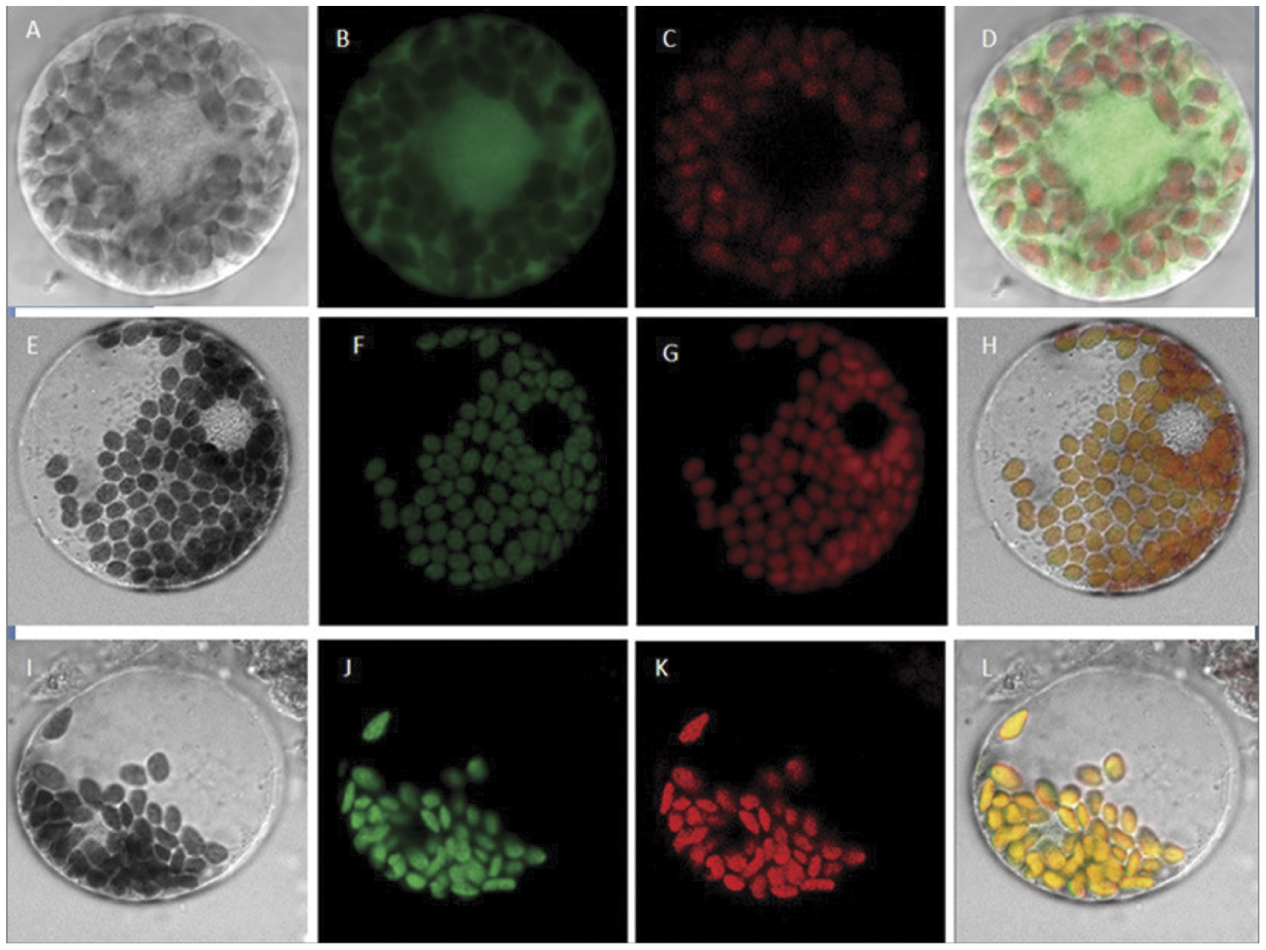
Subcellular localization of W69 and W106 proteins. The wheat W69 and W106 GFP fusion proteins localize to the chloroplasts in transiently transformed wheat leaf cells. After 12ﬂuorescence was detected. (A–D) control (16318hGFP) fluorescence detection; (E–H) W69-GFP fusion protein fluorescence detection; (I–L) W106-GFP fusion protein fluorescence detection. (A, E and I) bright field; (B, F and J) confocal ﬂuorescence; (C, G and K) chloroplast autoﬂuorescence; (D, H and L) overlay images. Scale bars  = 10 μm. Subcellular localization analysis was carried out four times with similar results.

GPX proteins in plants were found in the cytosol, chloroplast, and mitochondria. The PSORT system (http://www.psort.org/) program for predicting subcellular localization, indicated that both W69 and W106 with a chloroplastic N-terminal transit peptide would most probably localize in chloroplasts. To investigate the biological activity of the W69 and W106 proteins, the full-length cDNAs of W69 and W106 were fused in frame with the green fluorescent protein (*hGFP*) gene under the control of the CaMV 35S promoter, and transferred into wheat mesophyll protoplasts. Fluorescence of both W69-GFP and W106-GFP was specifically detected in chloroplasts, whereas the control expressing hGFP alone showed fluorescence distributed throughout the protoplasts (Fig. 5). Given their localization in chloroplasts, possible roles for W69 and W106 would be in protection of chloroplasts against oxidative damage induced by photosynthesis or external stresses [3].

### Improved salt tolerance in transgenic *Arabidopsis*

*GPX* genes are important in detoxification of many cellular degradation products formed during oxidative stress. Additionally, GPXs could impart stress tolerance through their peroxidase activities [45]. To further investigate the biological functions of the two GPXs, two T3 generation transgenic *Arabidopsis* lines were chosen to examine effects on germination and growth under salt stress. Transgenic and wild-type *Arabidopsis* seeds were grown on MS medium for 3 d at 22°C, and then transferred on MS medium containing different NaCl concentrations (50–200 mM).

**Figure 6:**
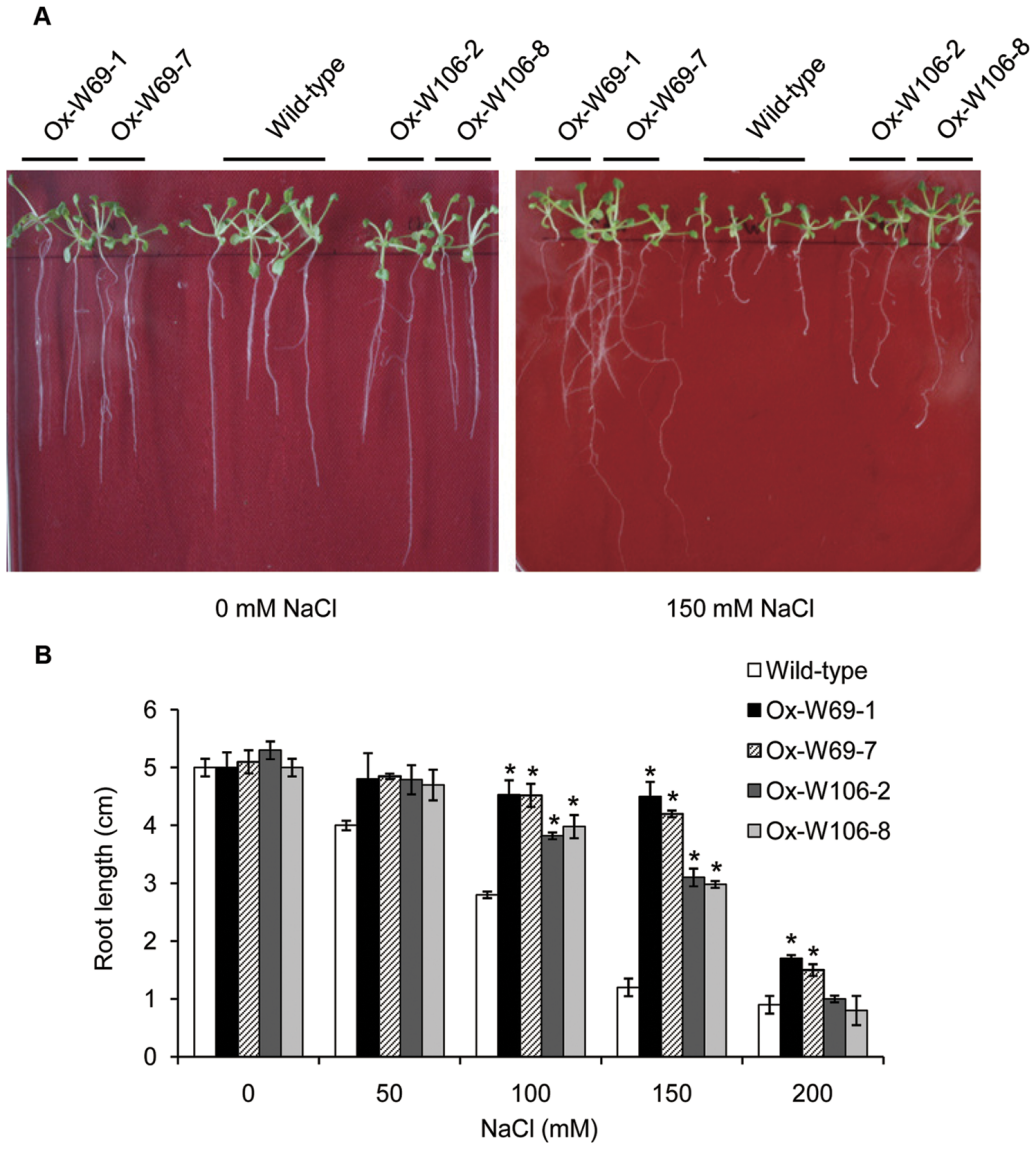
Responses of the wild-type and transgenic plants to salt treatment. (A) Phenotypes of wild-type, W69 and W106 seedlings under NaCl treatment. Three-day-old seedlings of four transgenic lines and wild-type *Arabidopsis* were planted on MS media with added NaCl (150 mM) for 15 d. (B) Comparative root lengths of wild-type, W69 and W106 seedlings grown on MS agar plates containing different concentrations of NaCl for 15 d after germination. Data are means ±SD of three independent experiments. Asterisks indicate a significant difference (*P<0.05; Student's t-test) relative to wild-type control.

Transgenic *W69* and *W106* seedlings had similar phenotypes to wild-type seedlings under normal conditions. As expected, salt stress reduced the growth of both transgenic seedlings and wild-type seedlings to some extent (Fig. 6). At 150 mM NaCl, transgenic plants remained green, whereas wild-type plants displayed chlorosis and growth inhibition after 15 days. The root lengths of W69-7 lines were almost 4-fold those of wild-type plants (Fig. 6A). Furthermore, transgenic *W69* seedlings were more tolerant of salt stress than seedlings of *W106* at different NaCl concentrations (Fig. 6B). In addition, the transgenic *Arabidopsis* plants showed higher germination rates than wild-type under high salt stress. Transgenic seeds germinated in up to 200 mM NaCl, whereas wild-type seeds failed to germinate under the same conditions (data not shown). Therefore, overexpression of *W69* and *W106* resulted in enhanced early tolerance to high salt stress.

We also examined tolerance to drought stress in the transgenic plants, but observed no detectable increases in tolerance (data not shown).

### Transgenic *Arabidopsis* has increased tolerance to H_2_O_2_

**Figure 7:**
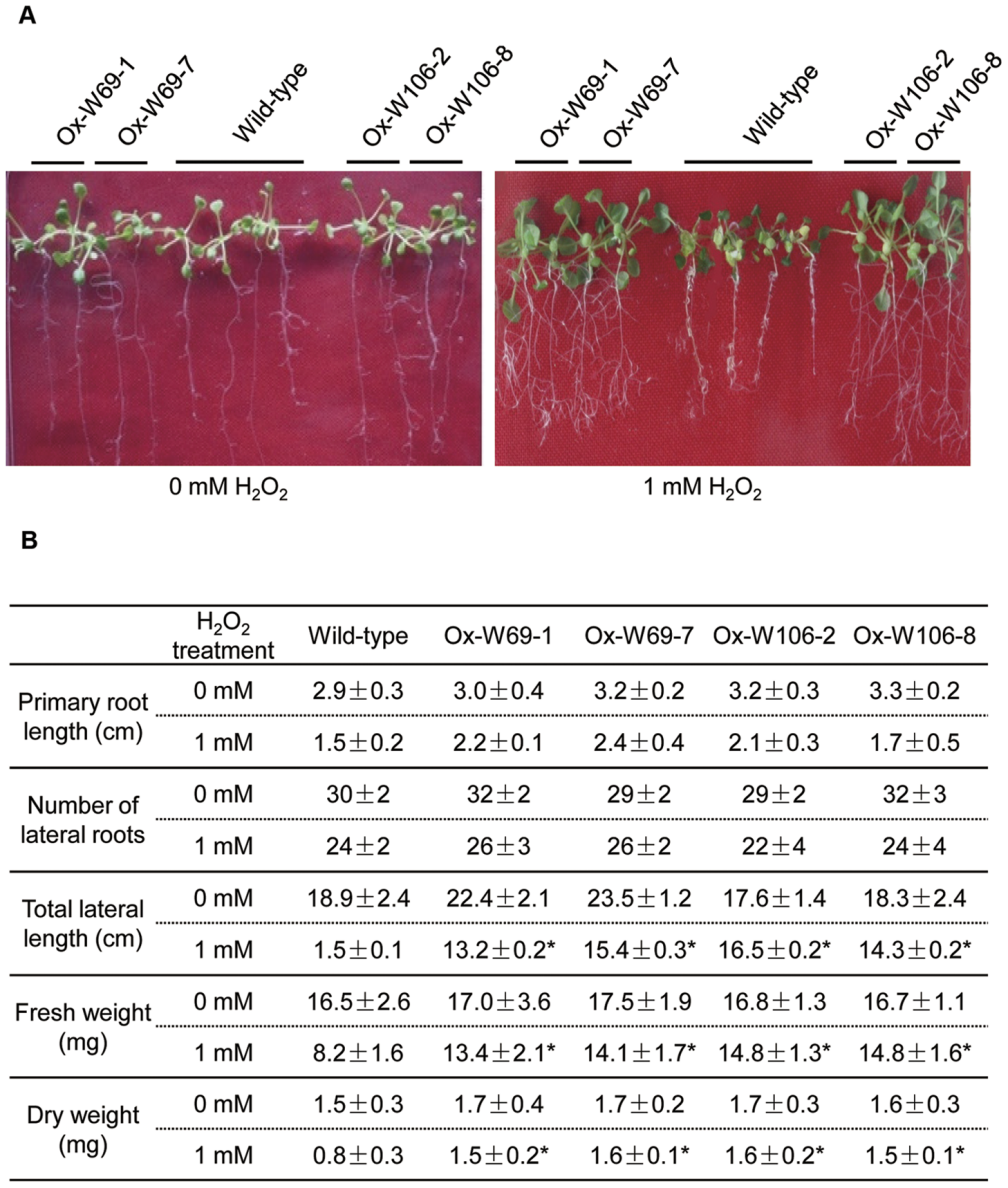
Responses of the wild-type and transgenic plants to H_2_O_2_ treatment. (A) Phenotypes of wild-type, W69 and W106 seedlings under H_2_O_2_ treatment. Three-day-old seedlings of four transgenic lines and wild-type *Arabidopsis* were planted on MS media with added H_2_O_2_ (1 mM) for 15 d after germination. (B) Effect of exogenous H_2_O_2_ on biomass production and root development of wild-type, W69 and W106 seedlings. After growing in MS media with added H_2_O_2_ (1 mM) for 15 d after germination, seedlings were collected and analysed. Data are given for the length of the primary root, lateral root number, total lateral root length, seedling fresh weight and dry weight. Data are means ±SD of three independent experiments. Asterisks indicate a significant difference (*P<0.05; Student's t-test) relative to wild-type control.

Three-day-old seedlings of transgenic and wild-type *Arabidopsis* were transferred to MS medium plates supplemented with 1 mM H_2_O_2_. At 15 d, the seedlings of *W69* and *W106* grew vigorously and were larger than wild-type seedlings. At 1 mM H_2_O_2_, the transgenics had longer primary roots than wild-type plants. There were also significant increases in the number and total lengths of the lateral roots compared to wild-type (Fig. 7A). As shown in Fig. 7B, H_2_O_2_ treatment significantly decreased the fresh and dry weights of transgenic and wild-type plants, but the transgenics were less affected by H_2_O_2_ treatment than wild-type plants. In addition, leaves of wild-type plants tended to become yellow during growth under H_2_O_2_ treatment whereas those of transgenic plants remained green (Fig. 7A). Thus the transgenic seedlings apparently had increased peroxide-scavenging capacity and enhanced tolerance to H_2_O_2_.

### Germination of W69 and W106-overexpressed seeds is insensitive to ABA

**Figure 8:**
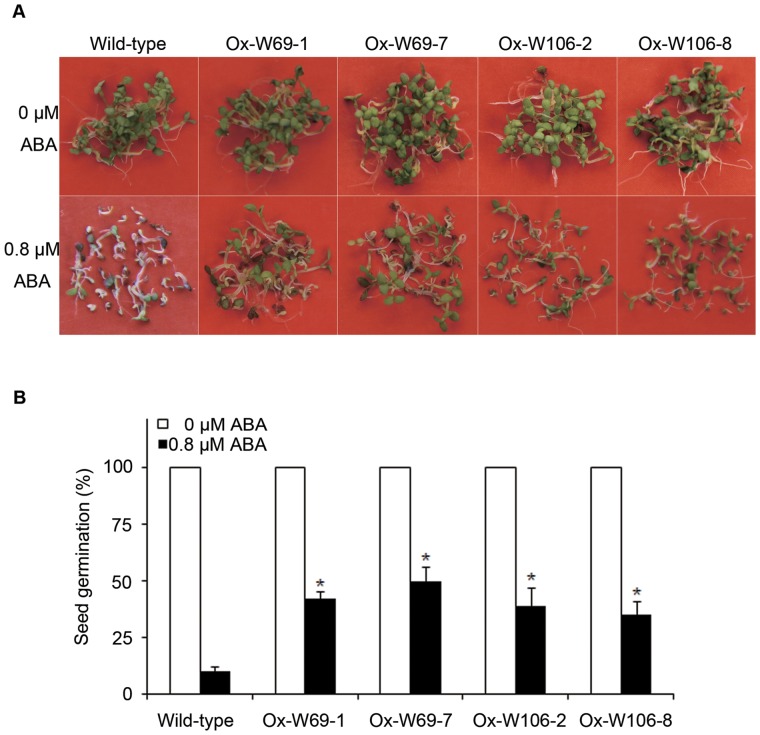
Responses of the wild-type and transgenic plants to ABA treatment. (A) Phenotypes of wild-type, W69 and W106 seedlings under ABA treatment. Seedlings of mixed transgenic lines and wild-type *Arabidopsis* were planted on MS media for 15 d. (B) Statistical analysis of seed germination on MS plates with added ABA (0.8 μM). 60 transgenic and wild-type seeds were plated on MS plates containing 0.8 μM ABA. Asterisks indicate a significant difference (P<0.05; Student's t-test) relative to wild-type control.

To investigate the effect of ABA on germination of *W69-* and *W106*-overexpressing seeds, we planted seeds on MS medium containing different ABA concentrations. As shown in Fig. 8A, there was no difference in seed germination between the wild-type and transgenic plants under normal conditions. At 0.8 μM ABA there was some inhibition of germination of both wild-type and transgenic seeds, but the latter were affected to a lesser extent. Approximately 42% of W69-7 overexpressed seeds and 39% of *W106*-2 overexpressed seeds developed cotyledons and became green compared with only 10% wild-type germination after 6 days (Fig. 8B). Addition of 1 μM ABA entirely arrested the emergence of cotyledons of both transgenic and wild-type seeds (Fig. 8B). These results indicated that *GPX* overexpression relieves the ABA-mediated inhibition during seed germination and early seedling growth.

### W69 and W106 displayed changed stress-responsive gene expressions

**Figure 9:**
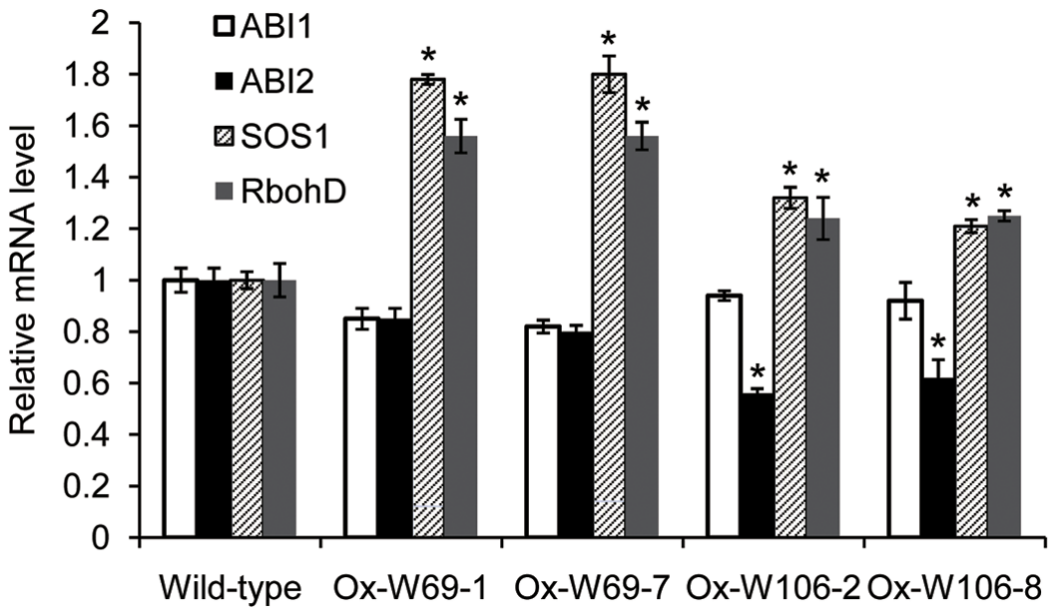
Expression effects of ABA- and stress-responsive genes in W69 and W106 *Arabidopsis* using Q-RT-PCR. Total RNA was isolated from leaves of 2-week-old *Arabidopsis* seedlings. The *actin2* gene was used as an internal reference. Relative expression of *W69* and *W106* were normalized to the transcript abundances in wild-type controls (normalized as 1). Error bars represent standard deviation among three biological replicates. Asterisks indicate a significant difference (P<0.05; Student's t-test) relative to wild-type control.

*SOS1*, *RbohD* and *ABI1/ABI2* are key regulators in response to salt, oxidative stress and ABA, respectively [39], [46]–[48]. To determine whether stress response pathways were affected by the GPX overexpression, we used Q-RT-PCR to analyse these genes in wild-type and transgenic *Arabidopsis*. Overexpression of *W69* and *W106* indeed changed the transcript levels of these marker genes. For example, *W69* activated *SOS1* and *RbohD* expression to levels about 1.8- and 1.5-fold higher, respectively, than those in wild-type. In contrast, the expression levels of *SOS1* and *RbohD* were only slightly higher in *W106* seedlings than in wild-type. Furthermore, *ABI1* and *ABI2* transcript levels in both transgenic lines appeared to be slightly lower than in wild-type (Fig. 9). These findings suggest that these GPXs may be signaling components in the ROS, ABA and salt-response pathways.

## Discussion

Animal GPXs are 20–22 kD monomer proteins, that can directly reduce phospholipid hydroperoxides, fatty acid hydroperoxides, and cholesterol hydroperoxides produced in peroxidized membranes [18]. Animal GPXs are considered crucial for protecting membranes from oxidative stress. Though GPX-like cDNAs had been found in a diversity of plants [22], [23], [38], [49]–[51], their physiological functions in plants had not been clearly resolved. Plant GPXs belong to a small GPX multigenic subfamily involved in response to various environmental stresses and had been considered to be the main defense strategy against oxidative membrane damage by detoxifying organic hydroperoxides as well as H_2_O_2_ [31], [52]–[55].

In the present study, we describe the identification and function of two wheat *GPX*s, designated *W69* and *W106*, that share relatively high levels of sequence similarity with other plant GPXs and human GPX04 (Fig. 1). Genomic sequences analysis of the two *GPX*s revealed that both comprise six exons and five introns. The nucleotide sequences and sizes of exons 2–5 of the isoenzymes genes were very similar, although the nucleotide sequences and sizes of the corresponding introns were highly divergent. These results provided evidence supporting tightly conserved exon-intron structures of *GPX* homologs across the plant kingdom [13]. In particular, there was a high degree of conservation among plant species in the lengths of exons 2–5, whereas the lengths of exons 1 and 6 were quite divergent [56]. The homologous human *HsGPX04* gene contains seven exons. Plant and mammalian *GPXs* have very divergent genomic organization patterns and analogous functions, reinforcing the hypothesis of an independent evolutionary scenario accompanying the structural diversification but functional similarity [13].

Salinity stress, mainly caused by NaCl and a serious threat to crop productivity, causes osmotic and ionic imbalances in cells [57]. On the other hand, salinity increases production and accumulation of ROS, which affects cell redox homeostasis and causes oxidative damage [58]. In the present work, we showed that overexpression of *W69* and *W106* increased the seed germination rate and boosted seedling growth in transgenic *Arabidopsis* plants during exposure to salt stress conditions (Fig. 6). In addition, exposure of wheat seedlings to NaCl or H_2_O_2_ induced rapid accumulation of *W69* and *W106* transcripts (Fig. 3). Therefore, it was likely that increased mRNA accumulation in *W69* and *W106* was an early response in ROS reduction or a manifestation of adjustment to environmental stress [59]. H_2_O_2_ is a central signaling molecule in stress and wounding responses, pathogen defense, and regulation of the cell cycle and cell death. Exogenous applications of H_2_O_2_ promote the formation and development of adventitious roots in seedlings, but a relatively higher concentrations H_2_O_2_ and longer times of exposure lead to inhibitory effects on rooting [60], [61]. Measurement of GPX activities showed that the two wheat GPXs can catalyze H_2_O_2_ or t-BHP *in vitro*. High level of GPX involved in the scavenging of H_2_O_2_ in plants helps to protect against H_2_O_2_-induced tissue injury and increases tolerance to oxidative damage induced by salt or ROS [62], [63]. Under exogenous H_2_O_2_ treatment, we found that transgenic plants became stronger than wild-type. In addition, the transgenic seedlings had greater total lateral root lengths than wild-type and improved tolerance to ROS stress (Fig. 7). W69 and W106 were located in the chloroplasts (Fig. 5), cell components with the highest production sites of ROS [64], [65]. These results indicated that overexpression of *W69* and *W106* conferred enhanced antioxidant capacity and protected plants against oxidative damage.

ABA is a phytohormone controlling seed dormancy and preventing seed germination during early embryogenesis. It also plays an essential role in stress response to drought, cold, and oxidative and osmotic stresses in the vegetative growth phase [66], [67]. Some reports indicate that ABA can trigger the production of H_2_O_2_ [68]–[70]. *Arabidopsis* seeds overexpressing *W69* and *W106* displayed higher germination rates than wild-type after ABA treatment, suggesting that transgenic seedlings have weakenedsABA control due to the scavenging effects of H_2_O_2_ (Fig. 8). Therefore, W69 and W106 might play an important role in the ABA signal transduction. It was reported that mutation of *AtGPX3* affected the expression of ABA-responsive genes and that the *AtGPX3* gene might act as an oxidative signal transducer in ABA and drought stress signaling [71]. Q-RT-PCR indicated that overexpression of *W69* and *W106* also affected the transcriptional levels of key regulator genes involved in ABA, H_2_O_2_ and salt signaling. This was in agreement with the important role of the GPX gene family in responding to environment stresses.

Analysis of upstream *cis*-regulating elements of *W69* and *W106* provided a way for us to understand how *GPX* expression was controlled. For instance, ABRE and other ABA-related elements (such as the DRE and DPBF binding sites) found in the promoters of *W69* and *W106* might be responsible for the responses of genes to ABA. The CRT/DRE and GT1GMSCAM4 elements present in the promoters of the two isoenzymes might be responsible for genetic responses to NaCl. More importantly, ARE, an antioxidant-responsive element in rat is also present in the promoters of the two isoenzymes, and might be related to genetic response to oxidative stress (Table 1). Collectively, *W69* and *W106* were not only participated directly in ROS signaling, but were also involved in ABA and salt signaling cascades.

In summary, overexpression of *W69* and *W106* in *Arabidopsis* seedlings provided protection from salinity stress during germination and seedling growth. This protective effect appeared to allow transgenic plants to retain high levels of metabolic activity and growth due to increased GPX expression and reduced levels of oxidative damage compared to wild-type seedlings. Our study should be beneficial for obtaining detailed insights into the complex function of plant GPXs, and for analyzing specialized physiological characteristics of this antioxidant system in other organisms [19]. The GPXs might be excellent candidates for genetical engineering of crop plants with improved salt tolerance.

## Materials and Methods

### Plant materials and stress treatments

Wheat (*T. aestivum* cv. Xiaobaimai) seedlings grown hydroponically at 25°C for 10 d were subjected to various abiotic stresses. Seedlings were exposed to air on filter paper for rapid induction of water stress. To mimic salinity and ABA treatments, roots were submerged in 100 mM NaCl or 100 µM ABA, respectively. For hydrogen peroxide treatment, seedlings were transferred to solutions containing 0.1 mM H_2_O_2_. Materials were collected at 0, 1, 2, 4, 16, 12, and 24 h after various treatments. Harvested plants were dropped immediately into liquid nitrogen and stored at –80°C for RNA extraction.

### General bioinformation

Putative amino acid sequences used in this study were from GenBank databases. Conserved motifs were investigated by multiple alignments using DNAMAN version 6.0. Phylogenetic trees were constructed with ClustalX using the neighbor-joining (NJ) method.

### Quantitative real-time PCR

Expression patterns for the two *GPX* genes were measured by Quantitative real-time PCR (Q-RT-PCR), using the ABI Prism 7300 system (Applied Biosystems, USA). Total RNA was extracted from young leaves of 2-week-old wheat plants using Trizol reagent according to the manufacturer's protocol (TianGen, Beijing, China) and DNase I digestions were applied (TaKaRa, Japan). First-strand cDNA was synthesized with AMV Reverse Transcriptase (TaKaRa, Japan). Each PCR was repeated three times in total volumes of 20 μl containing primer (4 μM), cDNA (40 ng) and 9 μl RealMasterMix (TianGen, Beijing, China). Quantification of the target gene expression under each stress was carried out by the relative 2−△△CT method [72]. The *actin* gene was used as an internal control for normalization of template cDNA. The gene-specific primers were as follows: *W69*, 5'- ACAGAGGATTCGGGAGGACT-3' and 5'- AGGAGGACCTTCCCCTTGTA-3'; *W106*, 5'-ACAAGGGGAAAGTCCTGCTT-3' and W106-RTR 5'-CTGGTTCCTGTCCAGCAAAT-3'; wheat *actin*, 5'- CTCCCTCACAACAACCGC-3' and 5'- TACCAGGAACTTCCATACCAAC-3'. Q-RT-PCR was also performed to analysis the expression of stress- and ABA-responsive genes, using 2-week-old transgenic and wild-type *Arabidopsis* seedlings. The *actin* gene was used as an internal control. The specific primers were as follows: *RbohD* (At5g47910), 5'-CCTCAACAACACCACCTCCT-3' and 5'-GTAAGAGGCCGTTGGAATCA-3'; *ABI1* (At4g26080), 5'-TGAAGAAGCGTGTGAGATGG-3' and 5'-CTGTATCGCCAGCTTTGACA-3' *ABI2* (At5g57050), 5’-TGCGGCGAGTAAAAGAAGAT-3' and 5'-TTCCTTTTTGCAAAGCCATC-3'; *SOS1* (At2g01980): 5'-ACCGGCAGATCTAATGAACG-3'and 5'-CTCCGCTACTGTCGATGTCA-3'; *Actin2* in *Arabidopsis*, 5'-GGTAACATTGTGCTCAGTGGTG-3' and 5'-C GACCTTAATCTTCATGCTGC-3'.

### Detection of enzyme activities of the GPX isoenzymes

The two GPX cDNAs were amplified by reverse transcription PCR (RT-PCR) using the primer sets W69-PET (5'-TACGGATCCATGGGGGCGTCCGAATCT-3' and 5'-TAGCTCGAGCTTCTGCGAGTCGGAAGATTCC-3') and W106-PET (5'-TACGGATCCAACATGGGTGCGGCAGAGT-3' and 5'-TAGCTCGAGAACCTCCAACAGCTTCTTGATG-3'). The PCR product was cut by *Bam*HI and *Xho*I and then subcloned into corresponding sites of pET-28a (Novagen) with a Histidine-tag at the N-terminus. The fusion plasmids and empty vector (PET-28a) were transformed into *E. coli* BL21 (DE3) strain. *E. coli* cells carrying pET-W69, pET-W106, and PET-28a were grown in 40 ml LB medium containing 50 mg/ml kanamycin at 37°C to D_600_  = 0.5 and induced with 0.8 mM isopropyl-1-thio-bD-galactopyranoside (IPTG) at 22°C. Cells were extracted at 4 h and were collected by centrifugation at 3000 g for 10 min, resuspended in pre-chilled sodium phosphate buffer (14 mM NaCl, 0.27 mM KCl, mM Na_2_HPO_4_, 0.18 mM KH_2_PO_4_, 1 mM EDTA-2Na and 1% polyvinylpyrrolidone, pH 7.0), and disrupted by sonication. His-tag fusion pET-W69/pET-W106/and/PET-28a proteins were purified using Ni-NTA Agarose (Qiagen, Tokyo, Japan) according to the manufacturer's instructions. Protein concentrations were quantified using the Bradford method. Purified proteins from each sample were loaded for sodium dodecyl sulfate polyacrylamide gel electrophoresis.

Enzyme activity was measured spectrophotometrically using a Glutathione Peroxidase Assay Kit (BioAssay systems, USA) with minor modifications. *E. coli* Trx and t-BHP were purchased from Sigma Aldrich (St. Louis, MO, USA). The reaction was started by addition of H_2_O_2_ or *t*-butyl hydroperoxide. The absorbance was immediately read at OD_340 nm_ and again at 4 min using a Perkin-Elmer Lambda 35 UV/VIS spectrometer (Perkin-Elmer Instruments, USA). The unit activity (U/L) is the amount of GPX that produces 1 μM of oxidized glutathione (GSSG) per min at pH 7.6 and room temperature.

### Transient expression of green fluorescent protein-fused GPXs

Green fluorescent protein (GFP)-fused GPXs, coexpression plasmids was constructed for the expression studies. The full open reading frames of the *W69* and *W106* genes were respectively cloned into 16318hGFP vectors, fused with the *GFP* reporter gene under the control of the 35S promoter [73]. The recombinant *W69-hGFP* and *W106-hGFP* fusion plasmids were transformed to common wheat mesophyll protoplasts by the PEG-mediated method [74]. The expression of fusion proteins was monitored after 12 h of incubation in a dark room, and images were captured under a laser scanning confocal microscope (Zeiss LSM700, Germany). GFP ﬂuorescence was collected in the 500–570 nm wavelength range. For chloroplast autoﬂuorescence, the wavelength range was 630–700 nm.

### Generation of transgenic *Arabidopsis* lines with *GPX* genes

Fragments of *W69* and *W106* were separately ligated into the modified pBI121 vector under control of the CaMV 35S promoter [75]. Kanamycin-resistant *Arabidopsis* transformants carrying *W69* or *W106* were generated using the vacuum infiltration method. Transformed plants were cultured on Murashige and Skoog (MS) medium containing 0.8% agar and 50 μM Kanamycin in a day/night regime of 16/8 h under white light (50 photons m^−1^s^−1^) at 22°C for 2 weeks and were then transferred to soil. The T3 generation seeds obtained were used for further analysis.

### Stress treatments

Seeds of transgenic (T3 generation) and wild-type *Arabidopsis* plants were grown on 10×10 cm MS agar plates. They were routinely kept for 3 days in the dark at 4°C to break dormancy and transferred to a tissue culture room under a day/night of 16/8 h under white light (with 50 photons m^−1^s^−1^) at 22°C for 3 d.

For salt treatment, 7-day-old seedlings were transferred to MS agar plates containing 150 mM NaCl for 7 days. For drought treatment, the seedlings were placed on MS agar plates with mannitol (100–200 mM) for 7 days. For H_2_O_2_ treatment, the plants were placed on MS agar plates supplemented with H_2_O_2_ (1–5 mM) for 7 days. For ABA treatment, the seeds of transgenic and wild-type *Arabidopsis* were grown on MS agar plates supplemented with 0.5–1 μM ABA for 7–14 days. Seeds were considered germinated when radicles had completely emerged from the seed coat.

The root lengths of *Arabidopsis* were measured with a ruler, and lateral roots were counted and measured with a dissection microscope. The fresh weight was measured on an analytical balance immediately after plant harvest. The samples used for dry weight determinations were measured after 48 h at 70°C.
